# The Hydrogenation of Crotonaldehyde on PdCu Single Atom Alloy Catalysts

**DOI:** 10.3390/nano13081434

**Published:** 2023-04-21

**Authors:** Mohammed J. Islam, Marta Granollers Mesa, Amin Osatiashtiani, Martin J. Taylor, Mark A. Isaacs, Georgios Kyriakou

**Affiliations:** 1 Energy & Bioproducts Research Institute (EBRI), College of Engineering and Physical Sciences, Aston University, Aston Triangle, Birmingham B4 7ET, UK; johirul5221@gmail.com (M.J.I.); a.osatiashtiani@aston.ac.uk (A.O.); 2 School of Engineering, Chemical Engineering, University of Hull, Cottingham Road, Hull HU6 7RX, UK; martin.taylor@hull.ac.uk; 3 Department of Chemistry, University College London, London WC1H 0AJ, UK; mark.isaacs@ucl.ac.uk; 4 HarwellXPS, Research Complex at Harwell, Rutherford Appleton Laboratory, Didcot OX11 0FA, UK; 5 Department of Chemical Engineering, University of Patras, Caratheodory 1, 265 04 Patras, Greece

**Keywords:** single atom alloy, single atom catalysts, PdCu, hydrogenation, crotonaldehyde

## Abstract

Recyclable PdCu single atom alloys supported on Al_2_O_3_ were applied to the selective hydrogenation of crotonaldehyde to elucidate the minimum number of Pd atoms required to facilitate the sustainable transformation of an α,β-unsaturated carbonyl molecule. It was found that, by diluting the Pd content of the alloy, the reaction activity of Cu nanoparticles can be accelerated, enabling more time for the cascade conversion of butanal to butanol. In addition, a significant increase in the conversion rate was observed, compared to bulk Cu/Al_2_O_3_ and Pd/Al_2_O_3_ catalysts when normalising for Cu and Pd content, respectively. The reaction selectivity over the single atom alloy catalysts was found to be primarily controlled by the Cu host surface, mainly leading to the formation of butanal but at a significantly higher rate than the monometallic Cu catalyst. Low quantities of crotyl alcohol were observed over all Cu-based catalysts but not for the Pd monometallic catalyst, suggesting that it may be a transient species converted immediately to butanol and or isomerized to butanal. These results demonstrate that fine-tuning the dilution of PdCu single atom alloy catalysts can leverage the activity and selectivity enhancement, and lead to cost-effective, sustainable, and atom-efficient alternatives to monometallic catalysts.

## 1. Introduction

Single atom catalysts (SACs) have emerged as an attractive new type of catalytic material which often combines the minimal use of high-cost precious metals with high activity and selectivity [1]. Over the past decade, these materials have been studied for a wide range of catalytic reactions, including hydrogenations [2,3,4,5,6,7], oxidations [8], and water–gas shift [9,10] reactions. A special class of SACs is single atom alloys (SAA), where trace amounts of atomically isolated metal atoms (typically a precious metal) reside on the surface of a host nanoparticle (often a lower cost metal). Such materials possess unique catalytic properties due to their electronic and geometric structure, which can facilitate or promote reactions that are often not possible with the host material exclusively [1]. This is the next stage in sustainable catalysis, where highly active materials can be synthesised at lower cost and applied, often exhibiting higher catalytic performance than classical ‘bulk’ catalysts. 

We have recently shown how Pd/Cu-SAAs supported on Al_2_O_3_ can be prepared utilizing the galvanic replacement method [11]. Based on this relatively simple synthetic protocol, individual Pd atomic entities were incorporated into the Cu host nanoparticle surface. Thorough characterisation of the materials with a wide range of techniques (including EXAFS, XANES, STEM, XPS, PXRD, and H_2_-TPR) confirmed the successful preparation of these materials. Furthermore, the Al_2_O_3_ supported Pd/Cu-SAA displays excellent performance towards the selective hydrogenation of furfural to furfuryl alcohol. In addition to the great benefit of atom efficiency of the Pd/Cu-SAA, high yields were also recorded with SAA catalyst performing nearly as well as the 1 wt.% Pd/Al_2_O_3_ monometallic catalyst and considerably better than the supported 1 wt.% Cu/Al_2_O_3_. 

**Figure 1:**
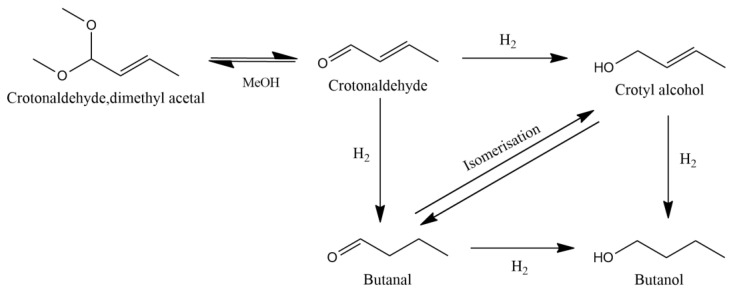
Reaction scheme for the transformation of crotonaldehyde.

In this work, we have utilized the same Pd/Cu-SAA materials to investigate the selective hydrogenation of crotonaldehyde, a smaller and relatively simpler unsaturated aldehyde as compared to furfural. The selective hydrogenation of α,β-unsaturated carbonyl compounds, such as crotonaldehyde, is a fundamentally interesting but also technically challenging catalytic task which finds applications in the production of fine chemicals and pharmaceuticals. The hydrogenation of crotonaldehyde, a planar π-conjugated molecule, has been previously studied both experimentally and theoretically using a range of catalysts in both the liquid and vapor phase [12,13,14,15,16,17,18,19]. The reaction can follow two main pathways, which are shown in Figure 1. Hydrogenating the C=C or the C=O bond in crotonaldehyde can lead to the formation of butanal or crotyl alcohol, respectively. Both these molecules can then be further hydrogenated to butanol via the reduction of their remaining unsaturated double bond, a product of major importance for the liquid fuel industry. It should be noted that the formation of crotyl alcohol is of particular interest. This is because the absence of a directing group, such as the furan ring in furfural, makes the selective hydrogenation of the aldehyde group comparatively more difficult [20]. Thermodynamics favours the hydrogenation of the C=C bond to form the saturated aldehyde by 35 kJ mol^−1^. Therefore, the formation of crotyl alcohol requires the use of suitable catalysts which can direct the selective hydrogenation of C=O bond, exclusively. However, in the liquid phase, and much the same for other aldehyde hydrogenation reactions, there is often a coupling reaction with the solvent itself generating an acetal species which is classically seen as acid catalysed [21]. 

A range of metal catalysts has been previously studied for the selective hydrogenation of crotonaldehyde in the literature, using Ag, Co, Cu, Ir, Mo, Nb, Ni, Pt, Pd, Re, Rh, Ru, and W [5,14,18,20,22,23,24,25,26,27,28,29]. Base-metals such as Ni and Cu [24,29,30] are reported to preferentially hydrogenate the C=C bonds, as the crotonaldehyde molecule typically adsorbs with a planar geometry on the surface. There has been significant interest in Cu-based catalysts [17,18,24,30] for such reactions, as it is inexpensive compared to platinum group metal catalysts in which steric [29] and electronic [17] modifications have been employed to promote perpendicular crotonaldehyde adsorption, such that only the C=O is hydrogenated. For example, adsorbed sulphur species have been used to cause the rehybridisation of the adsorbed reactant, resulting in the weakening of the intermolecular bonding and tilting of the C=C and C=O groups relative to the surface [17]. Thus, this favours the unsaturated alcohol formation when using Cu-based catalysts. While Pt-based catalysts are reported to show high crotyl alcohol selectivity, due to a similar tilting of the reactant as the coverage of the crotonaldehyde increases [31], Pd catalysts have been found to be generally unselective, where the C=C and C=O are both hydrogenated. Campo et al. [23] reported that monometallic Pd catalysts were unselective towards crotyl alcohol. Modification of the Pd with oxidic species [23] or Ni as a bimetallic [22] had a minimal effect on changing the selectivity towards crotyl alcohol or the synergistic behaviour. However, it has been reported earlier that the lack of selectivity towards crotyl alcohol may be the result of the isomerisation of crotyl alcohol to butanal, promoted by Al_2_O_3_, Cu, Pd, and Ni materials [32,33,34]. Recently, various single-atom catalysts have been utilised to develop better, atom-efficient and selective materials for the hydrogenation of crotonaldehyde. A Pt_1_/MoC catalyst synthesized by Qingyuan et al. [35] reported a 4 time increase in TOF (1216 h^−1^) versus a monometallic 1% Pt/MoC (299 h^−1^). However, the reported selectivity was found to be equally distributed across the three possible hydrogenation products (butanal, crotyl alcohol, and butanol). In contrast, another Rh_1_/MoS_2_ SAC synthesised by Yang et al. [5] reported a 100% crotyl alcohol selectivity. They attributed the incredible selectivity to “pocket”-like active sites, which promoted vertical adsorption of crotonaldehyde and hydrogenation of only the C=O bond through steric effects. However, these sites lacked activity (TOF = 64.7 h^−1^) compared to the Qingyuan Pt_1_/MoC catalyst. 

The use of a Pd/Cu-SAA catalyst for the hydrogenation of crotonaldehyde is particularly interesting for a variety of reasons. The presence of trace amounts of Pd single atomic entities is expected to have an impact on the reaction rate, as it provides an alternative pathway for the dissociation of H_2_ molecules and the spillover of hydrogen atoms across the catalyst surface. However, the product selectivity of the reaction depends strongly on the type of metal used and various experimental parameters, with Cu nanoparticles being selective towards the hydrogenation of the C=C bond, while Pd nanoparticles being relatively unselective to which bond is hydrogenated. In the special case of a Pd/Cu-SAA, the critically low concentration of Pd present in the catalyst, once dispersed, exists in the form of single atom sites on the Cu surface, and therefore, the selectivity is expected to be controlled mostly by the Cu host nanoparticles (i.e., the dominant metal on the surface of the SAA). This present work will shed light on the active sites involved in the hydrogenation of crotonaldehyde and the action of the Pd single atomic entities in the catalytic reaction. 

## 2. Materials and Methods

### 2.1. Catalyst Synthesis and Characterisation

The synthesis and characterisation of the catalysts used in the present study were described in our earlier work [11]. Briefly, the SAA catalysts were synthesized using the galvanic replacement method in which Cu atoms from a 1 wt.% Cu/Al_2_O_3_ (Cu_100_) catalyst are replaced by Pd atoms on host Cu nanoparticles. The Cu/Al_2_O_3_ catalyst was prepared colloidally [36] and deposited on a nanophase-alumina support. Similarly, the Pd/Al_2_O_3_ (Pd_100_) catalyst was also synthesized colloidally [37,38] and deposited on the same nanophase alumina support. Table 1 summarizes the catalysts used in the present study and their major physicochemical properties. Metal contents were determined by Inductively Coupled Plasma Optical Emission Spectroscopy (ICP-OES) using a Thermo Scientific iCAP 7400 Duo in both axial (increases sensitivity at low concentrations) and radial modes for Pd and Cu content, respectively. X-ray absorption spectra were collected at B18 XAS beamline at Diamond Light Source, United Kingdom in fluorescence mode, scanning in the energy range from −200 to 800 eV relative to the Pd K-edge (24,350 eV). X-ray photoelectron spectroscopy (XPS) and X-ray excited Auger spectroscopy (XAES) spectra were acquired on a Kratos AXIS Supra spectrometer equipped with a charge neutraliser and monochromated Al Kα excitation source (1486.7 eV) with energies referenced to adventitious carbon at 284.8 eV using CasaXPS version 2.3.19PR1.0 and a U 2 Tougaard background. Bulk elemental analysis showed that trace amounts of Pd were incorporated into the Cu nanoparticles, with EXAFS determining the Pd species were atomically dispersed on the Cu nanoparticles (no Pd-Pd interactions). Thus, this confirmed the formation of single-atom catalysts, specifically, single-atom alloy catalysts. EXAFS and XPS characterisation differentiated the catalysts by suggesting that the Pd_1_Cu_216_ catalyst had a larger ratio of Pd atoms remaining on the surface of the nanoparticle than the higher Pd loaded Pd_1_Cu_53_ catalyst. Additional characterisation of the materials shown in Table 1 can be found in our earlier work [11]. Note that the Pd dispersion could not be determined for Pd_1_Cu_234_ due to the low intensity of the Pd 3d signal during XPS analysis (below the detection limit). Additionally, the high dilution of the Pd content in this catalyst did not lead to any meaningful EXAFS data; therefore, an EXAFS designation was not added to Table 1. However, since the more concentrated Pd_1_Cu_53_ and Pd_1_Cu_216_ materials were designated as SAAs based on EXAFS, it is appropriate to consider Pd_1_Cu_234_ also as a SAA.

### 2.2. Catalytic Testing

In situ reduction and catalytic reactions were carried out in a Hel DigiCAT high-pressure parallel reactor system, housing a bank of two 50 mL stainless steel reactor vessels. The reaction conditions used in this work were similar to those used in earlier studies in the literature [4,11,21,39] and provided a benchmark to compare the catalytic activity and make correlations with the nanoparticle structure, catalyst composition and recyclability. The vessels were loaded with ∼10 mg of the catalyst, heated under flowing H_2_ to 300 °C at 5 °C min^−1^ and held for 0.5 h before cooling to room temperature under flowing H_2_. The autoclaves were subsequently sealed and purged with H_2_ to prevent/minimize any oxidation of the catalyst. Reaction solutions consisting of crotonaldehyde (0.02 M, Sigma Aldrich, ≥99.5%) and internal standard dioxane (0.02 M, anhydrous, Sigma Aldrich, 99.8%) in methanol (MeOH) as the solvent were sonicated for 5 min in an ultrasonic bath to ensure solubility and to remove dissolved gases. Under H_2_ flow, 10 mL was injected into each reactor. The reactors were degassed for 10 min under H_2_ flow (1.5 bar, BOC, 99.995%), followed by sealing and heating to 50 °C and stirring at 600 rpm. The reactions were all carried out for 7 h at 50 °C before being cooled and depressurised to atmospheric pressure. The external mass transfer was assessed by changing the stirring speed from 600 to 900 rpm and no appreciable differences were detected within this range. Aliquots of the reaction mixture (0.2 mL) were taken, filtered and analysed with a 1:4 dilution in MeOH on a Shimadzu GC-2010 Plus equipped with a flame ionisation detector (FID) and a Zebron ZB-WAX capillary column (5%-phenyl-95%-dimethylpolysiloxane, 30 m × 0.53 mm × 1.50 μm). The concentration of the products was determined through the normalisation of the individual peak areas with the internal standard, as well as the use of 5-point calibration standards of the pure compounds. Reaction mixtures were further qualitatively analysed by GCMS on a Shimadzu GCMS-QP2010 SE. 

## 3. Results and Discussion

**Figure 2:**
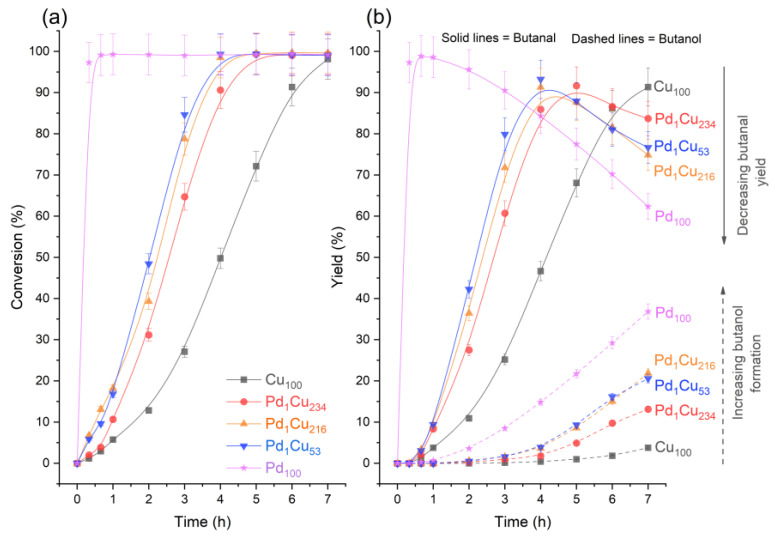
Reaction profiles for the hydrogenation of crotonaldehyde. (**a**) crotonaldehyde conversion and (**b**) yield of major products across monometallic and bimetallic catalysts. Reaction conditions: 7 h, 50 °C, 1.5 bar, 600 RPM, and 10 mg of catalyst.

The performance of the monometallic Cu and Pd catalysts as well as the Pd augmented Cu-based SAA catalysts were explored for the catalytic hydrogenation of crotonaldehyde at 50 °C with 1.5 bar of H_2_. The results are summarized in Table 2 and Figure 2. The transformation of crotonaldehyde can follow various pathways which have been summarised in Figure 1. In the absence of any Cu/Pd (bare support or in the absence of any solid), hydrogenation reactions were not observed (Table 2). However, coupling between the crotonaldehyde and MeOH in the form of acetalisation was observed for both the bare support and blank reaction. In the presence of the Cu monometallic catalyst, butanal is the main hydrogenation product with a selectivity of ~93% with minor quantities of butanol and crotyl alcohol being also observed with a selectivity of 3.9% and 1.5%, respectively. In marked contrast, the Pd monometallic catalyst produces butanal with a much lower selectivity (62.8%), while butanol forms with a selectivity of 37.2%. Notably, crotyl alcohol was not observed with the Pd monometallic catalyst. 

Figure 2a illustrates that all the catalysts can achieve near 100% crotonaldehyde conversion in 7 h at 50 °C and 1.5 bar H_2_, albeit through different reaction pathways. The conversion profiles are similar to those shown for furfural with these SAA catalysts, but with higher activity [11]. This means that irrespective of the differences in physical structure between furfural and crotonaldehyde, the SAAs remain selective across both molecules. Also consistent with this previous work, there was a catalyst induction period of 0.67–1 h for all the Cu-based catalysts where the conversion is suppressed; this behaviour is assumed to be due to the limited catalytically available hydrogen at the beginning of the reaction, either through the formation of surface oxide (from O_2_ contamination) or Cu’s inability to adequately chemisorb hydrogen. The presence of Pd with the SAA catalysts appears to lessen these effects, and it is in stark contrast to the monometallic Pd_100_ catalyst, which is lacking such an induction period due to its increased resistance to oxidation, higher reducibility and likely again due to the extended Pd surface to store hydrogen as β-hydride species during the in situ reduction treatment [40,41,42,43].

Adding trace amounts of Pd to the Cu nanoparticles has a significant impact on the catalytic performance as compared to the Cu_100_ host catalyst (Figure 2). The PdCu-SAAs display a remarkable improvement in terms of crotonaldehyde conversion (Figure 2a), as compared to the monometallic Cu catalyst, reaching nearly 100% conversion in much shorter reaction times. Consequently, the PdCu-SAA catalysts were able to hydrogenate further the butanal C=O bonds, decreasing the yield of butanal and increasing the formation of butanol, as shown in Figure 2b. It was also noticeable that PdCu-SAA differed from the Pd_100_ catalytic performance, as the former were reaching a maximum butanal yield of ~92%, while the Pd_100_ showed a maximum butanal yield of ~99% after 1 h of reaction. Additionally, the lack of crotyl alcohol and acetal in the Pd_100_ catalyst demonstrated the different behaviour of PdCu-SAA compared to monometallic Pd. 

Table 2 and Figure 2 clearly show that all the PdCu-SAA catalysts initially promote the hydrogenation of the C=C bond to butanal with minimal selectivity to crotyl alcohol (C=O bond hydrogenation). This observation is in marked contrast to furfural hydrogenation on Cu based catalysts, which leads to the facile hydrogenation of the C=O bond due to the furan ring being electronically repulsed from the surface [11]. The perpendicular η ^1^(O) conformation of furfural [39] leads to the selective hydrogenation of the C=O bond. However, in the case of crotonaldehyde, NEXAFS experiments by Chiu et al. [24] suggest that on Cu(111) surfaces, crotonaldehyde adopts a planar adsorption geometry on the surface, leading to low selectivity towards the C=O bond hydrogenation. Since the PdCu-SAA catalysts in the present work derive their selectivity from the binding modes of crotonaldehyde on the host nanoparticle’s surface, the preference for C=C hydrogenation is understandable. Interestingly, DFT calculations on Pd(111) surfaces have shown that crotonaldehyde follows a similar binding mode, parallel to the surface via the C=C and C=O bonds. As a result, these conformations promoted either full hydrogenation to butane or partially to butanal [15].

**Figure 3:**
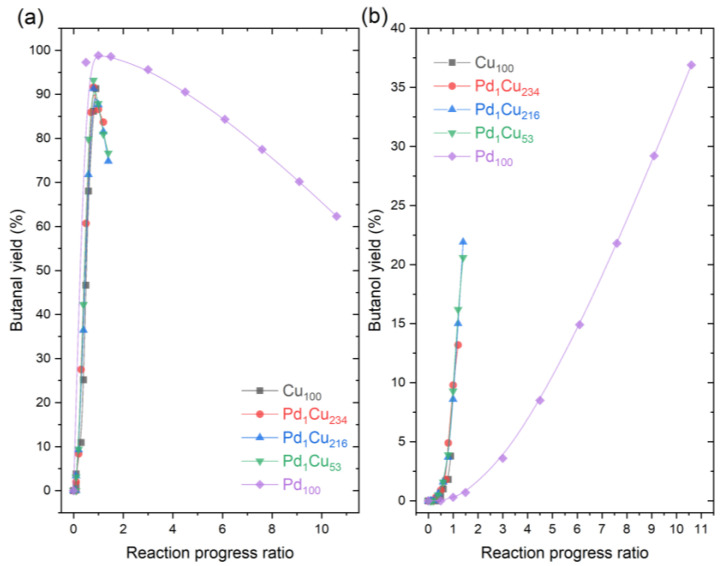
Yields vs. Reaction progress ratio for the hydrogenation of crotonaldehyde. (**a**) Yield of butanal; (**b**) Yield of butanol. Reaction conditions: 7 h, 50 °C, 1.5 bar H_2_, 600 RPM, and 10 mg of catalyst.

To further corroborate if the selectivity in PdCu-SAA is controlled by the host Cu nanoparticle, the yields of butanal and butanol from Figure 2b were represented versus the reaction progress ratio (RPR), in order to normalise and compare yields at similar extents of the reaction (Figure 3). The reaction progress ratios in the *x*-axis of Figure 3 were calculated using reaction time over time to reach 99% crotonaldehyde conversion. When the value of RPR equals 1 is when 99% conversion has been achieved for the first time in the experiment, and any values over 1 reflect any further evolution in composition generated by other reactions. 

Figure 3a shows that the maximum yield of butanal is achieved at reaction progress ratios of 1 for all catalysts, with a different maximum for Pd_100_ (~98%) compared to the other catalyst (~93%). This indicates that, while there is crotonaldehyde to react, the butanal yield is favoured. Beyond that point, the butanal yield decreases and the butanol yield increases. This suggests that once the hydrogen is not used for the conversion of crotonaldehyde, the hydrogen is spent on hydrogenating the butanal. Both Figure 3a and Figure 3b show that PdCu-SAA follow the same evolution pathway with respect to the reaction extent as Cu_100_, and the only difference is that they enable further progress of the hydrogenation of butanal to butanol. The data also highlight the deviation of the Pd_100_ with respect to the other catalysts, as the Pd_100_ showed higher yields of butanal and lower yields of butanol throughout the entire extent of the reaction. This is expected, as Pd is known to be a relatively poor catalyst for the hydrogenation of the C=O bonds, especially in the presence of large Pd particles and low density of edge sites [14]. On the contrary, the Cu_100_ and PdCu-SAA catalysts show more capability to hydrogenate the C=O bond even before reaching 100% conversion of crotonaldehyde. This is evidenced by Figure 3b, where the yields of butanol increased at earlier rate progression ratios, and by the presence of crotyl alcohol in both Cu_100_ and PdCu-SAA catalysts. Therefore, this confirms that the main function of diluted Pd in the Cu nanoparticle host surface is to accelerate the reaction rather than changing the reaction pathway, very likely by lowering the hydrogen dissociative adsorption barrier and subsequent spillover onto the Cu surface [44]. 

**Figure 4:**
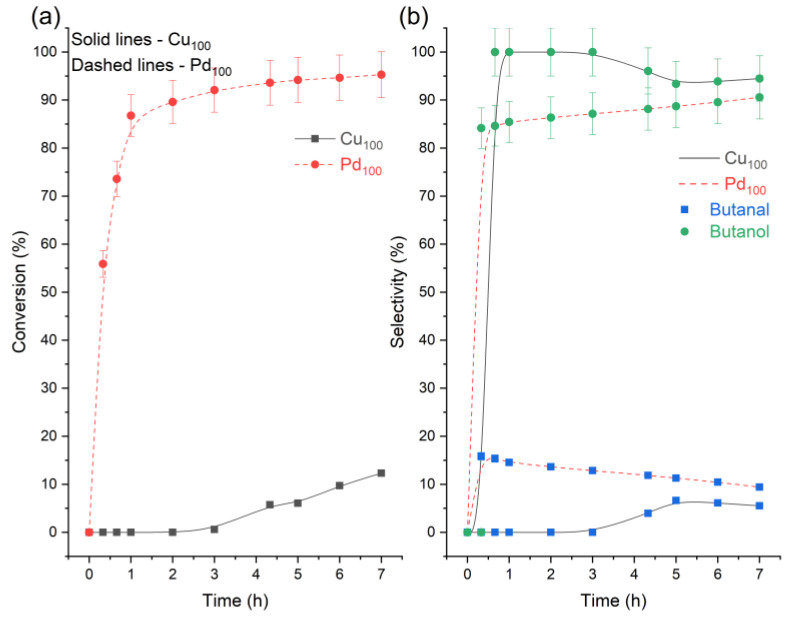
Reaction profiles for the hydrogenation of crotyl alcohol. (**a**) crotyl alcohol conversion and (**b**) product selectivity across the monometallic catalysts. Reaction conditions: 7 h, 50 °C, 1.5 bar H_2_, 600 RPM, and 10 mg of catalyst.

Analysing the yields, it can be speculated that in all the catalytic systems tested, the reaction proceeds largely by the hydrogenation of crotonaldehyde to butanal (via the C=C bond) and then the subsequent reduction of butanal to butanol (via the C=O bond) [14]. However, past studies [32,33,34] suggest the mechanism may be more complex as butanal may also be formed from crotyl alcohol acting as an intermediate, isomerising into the saturated aldehyde (Figure 1). It has been reported for Cu-Cr catalysts that the selectivity for forming crotyl alcohol is lower than for the isomerisation reaction, so the majority of crotyl alcohol is transformed into butanal [33]. To understand this reaction better, further experiments were conducted replacing the reactant crotonaldehyde with crotyl alcohol under the same conditions (Table 3 and Figure 4). Both monometallic catalysts were found to be able to isomerise crotyl alcohol to butanal but with a major selectivity towards crotyl alcohol’s hydrogenation to butanol. The data also provide some evidence on why the Pd_100_ catalyst is entirely unselective towards crotyl alcohol, as it is quickly either converted to butanol or butanal. However, since the crotyl alcohol intermediate is not detected, it is likely crotyl alcohol is not initially formed.

**Table 3:** Summary of the catalytic data for the hydrogenation of crotyl alcohol using monometallic Pd and Cu catalysts. Reaction conditions: 7 h, 50 °C, 1.5 bar H_2_, 600 RPM, and 10 mg of catalyst.

Catalyst	Conversion (%)	Butanal S (%)	Butanol S (%)	Carbon Balance (%)
Cu_100_	12.3 ± 0.6	5.5 ± 0.3	94.5 ± 4.8	100 ± 5.0
Pd_100_	95.3 ± 4.8	9.4 ± 0.5	90.6 ± 4.5	92.1± 4.6

**Figure 5:**
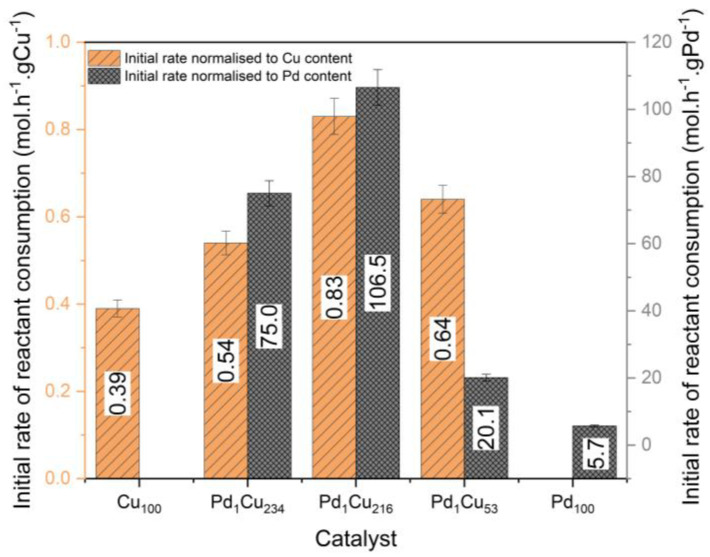
Initial rate of crotonaldehyde consumption normalised to Cu and Pd content for the hydrogenation of crotonaldehyde. The initial rate was determined after the induction period for the Cu-based catalysts. Reaction conditions: 7 h, 50 °C, 1.5 bar H_2_, 600 RPM, and 10 mg of catalyst.

Mirroring our previous work on furfural with the PdCu-SAA [11], the dilute palladium materials are considerably more active in terms of their atom efficiency compared to their monometallic (Cu and Pd) counterparts (Figure 5). Principally, the Pd_1_Cu_216_ catalyst demonstrates a nineteen-fold increase in activity compared to the monometallic Pd_100_ catalyst, which is due to the lack of spectator Pd atoms in the nanoparticle’s bulk. Thus, demonstrating the synergistic behaviour of Pd atoms on Cu surfaces for the hydrogenation of crotonaldehyde. The Pd_1_Cu_234_ and Pd_1_Cu_216_ catalysts show that adding trace amounts of Pd have the greatest effect, while further Pd addition has a diminishing return in the activity, indicating a minimum necessary number of Pd active sites required to both activate hydrogen and direct adsorption of the crotonaldehyde. It is proposed that this trend is due to the Pd atoms being inaccessible as hydrogen dissociation entry sites since the EXAFS suggests a considerable quantity of the Pd atoms have diffused under the surface of the nanoparticle for the Pd_1_Cu_53_ catalyst.

**Figure 6:**
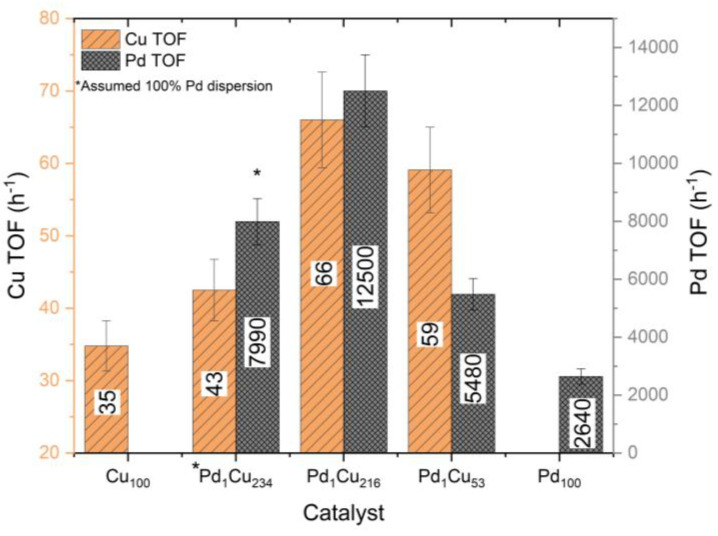
Turnover frequency of both Cu and Pd surface atoms for the catalysts determined from the XPS calculated dispersion for the hydrogenation of crotonaldehyde. Reaction conditions: 7 h, 50 °C, 1.5 bar H_2_, 600 RPM, and 10 mg of catalyst.

Turnover frequencies (TOFs) were determined in Figure 6 to identify whether the synergetic effect of the atomically dispersed Pd atoms were due to the difference in the dispersion of the catalysts. TOFs indicate the introduction of the atomically dispersed Pd atoms augments the catalytic activity of the surface Cu sites. For example, the optimal Pd_1_Cu_216_ catalyst boosts Cu TOFs by ~90%. Further raising the Pd loading with the Pd_1_Cu_53_ comes with diminishing returns as the TOF and normalised initial rates are not promoted. Comparing TOFs of the monometallic Pd_100_ catalyst to a SAA catalyst shows that the atomically dispersed Pd sites are ~373% more catalytically active than the equivalent surface sites found on Pd nanoparticles. Furthermore, drastic increases in the Pd loading with the Pd_1_Cu_53_ catalyst appear to begin to render the Pd sites less effective, possibly changing their properties to be more like that of a bulk monometallic Pd catalyst. It should be noted that due to the inability to determine the Pd dispersion for the Pd_1_Cu_234_ catalyst (lack of Pd 3d signal), it was assumed to be 100% dispersed for this TOF calculation.

**Figure 7:**
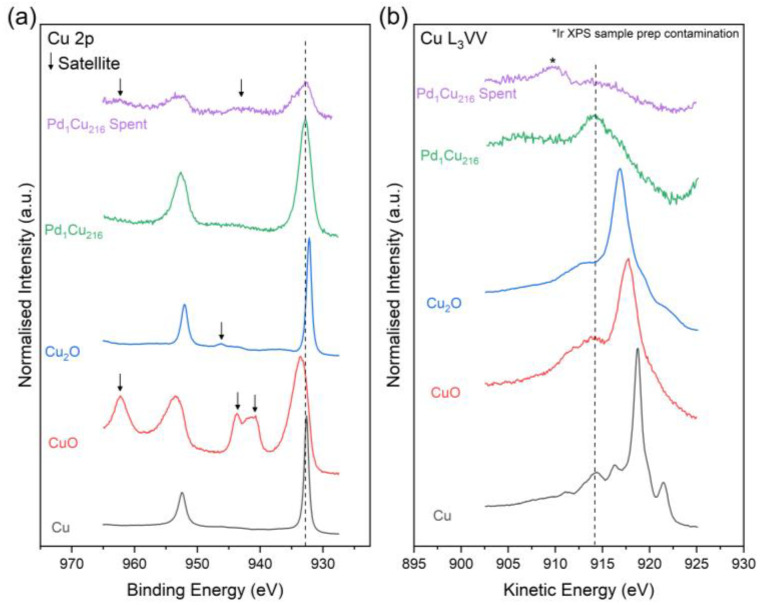
High-resolution stacked XPS and XAES spectra of the (**a**) Cu 2p and (**b**) Cu L_3_VV regions for the spent and unused Pd_1_Cu_216_ catalyst together with the CuO Cu_2_O and Cu standards [11].

Comparing the XPS spectra of the spent and unused Pd_1_Cu_216_ catalysts (Table 4 and Figure 7) shows that, like for furfural hydrogenation, as previously reported [11], the spent catalyst is somewhat oxidised to CuO. This is observed by the presence of the shake-up satellites and the broadening of the Cu 2p_3/2_ transition to higher energies. Supporting this, the surface compositional analysis in Table 4 confirms the Cu species are largely in their Cu^2+^ oxidation state (58.7%). It should be noted here that recovery of the catalyst from the reaction mixture involves centrifugation, washing and drying in air at 60 °C which, unavoidably, lead to the oxidation of the Cu, as observed in the XPS analysis. The spectrum of the spent material was also found to be severely attenuated compared to the unused catalyst, which is likely due to the small amount of catalyst used and recovered from the catalytic testing. Consequently, the position of the Cu L_3_VV Auger transition cannot accurately be determined (Figure 7). The XPS calculated Cu dispersion values show minimal loss (~4.6%) in Cu dispersion through nanoparticle sintering, which is within error. As mentioned previously, Pd designation was limited due to the Pd 3d signal being below the detection level of the instrument. 

**Table 4:** XPS data summary. Binding energies for the Cu 2p_3/2_ and kinetic energies of the Auger Cu L_3_VV transitions, surface composition and XPS calculated Cu dispersion for the unused and spent catalysts.

Sample	State	Cu 2p_3/2_ (eV)	L_3_VV (eV)	Cu^0^ + Cu^+^ (%)	Cu^2+^ (%)	Cu Dispersion (%)
Pd_1_Cu_216_	Fresh	932.83	914.24	97.6	2.4	80.3 ± 8.0
Pd_1_Cu_216_	Spent	932.73	N/A	41.3	58.7	61.5 ± 6.2
CuO	-	933.62	917.78	-	-	-
Cu_2_O	-	932.29	916.70	-	-	-
Cu *	-	932.63	918.75	-	-	-

* Cu 2p_3/2_ was calibrated to the ISO standard of 932.63 eV.

The recyclability of the catalysts was also investigated. The catalysts were recovered after the reaction via centrifugation, followed by washing with methanol. Once dried, they were retested at 75% scale. Table 5 shows that the conversion and selectivity of the catalysts were, within error, minimally affected after reuse, suggesting no loss of active metal. 

## 4. Conclusions

In this work, the hydrogenation of crotonaldehyde was studied on a series of Al_2_O_3_ supported Pd/Cu SAA catalysts. The catalytic results were compared with those obtained with Al_2_O_3_ supported Cu and Pd monometallic catalysts. The catalytic properties of the Cu and Pd monometallic catalysts show substantial differences in terms of both activity and selectivity. Pd was found to be more active than Cu but relatively unselective towards the hydrogenation of the C=C and C = O. Cu was found to primarily lead to the formation of butanal. The addition of trace amounts of Pd (0.0067 wt.%) on the Cu surface was found to improve the normalised catalytic activity (with respect to the individual metal loading) by nineteen-fold and two-fold when compared to the monometallic Pd and Cu catalysts, respectively. The selectivity of the catalytic reaction over the SAAs was found to be primarily controlled by the Cu host surface, predominantly leading to butanal. However, a prolonged reaction time over the SAA catalysts was found to increase the yield of butanol up to 4.8 times, as compared to the monometallic Cu catalyst. The selectivity of all Cu-based catalysts towards crotyl alcohol was relatively low (<2%) as Cu surfaces can isomerize this molecule to butanal and subsequently hydrogenate it to butanol.

## Figures and Tables

**Table 1:** Summary of the characterisation data for the supported catalysts [11].

Catalyst	Pd Loading (wt.%)	Cu Loading (wt.%)	Pd:Cu Atomic Ratio	Particle Size (nm)	Cu Dispersion (%)	Pd Dispersion (%)	EXAFS Designation
Cu_100_	-	0.9403 ± 0.0267	-	2.7 ± 0.7	71.0 ± 7.1	-	-
Pd_1_Cu_234_	0.0064 ± 0.0006	0.8947 ± 0.0253	1: 234	2.6 ± 0.7	79.9 ± 8.0	N/A	-
Pd_1_Cu_216_	0.0067 ± 0.0006	0.8599 ± 0.0262	1: 216	2.0 ± 0.6	80.3 ± 8.0	90.9 ± 9.1	SAA
Pd_1_Cu_53_	0.0296 ± 0.0022	0.9296 ± 0.0232	1: 53	7.0 ± 4.4	68.9 ± 6.9	41.7 ± 4.2	SAA
Pd_100_	0.8882 ± 0.0529	-	-	5.1 ± 2.7	-	22.9 ± 2.3	-

**Table 2:** Summary of the catalytic conversion and selectivity (S) for the hydrogenation of crotonaldehyde using Pd/Cu catalysts. Reaction conditions: 7 h, 50 °C, 1.5 bar H_2_, 600 RPM, and 10 mg of catalyst.

Catalyst	Conversion (%)	Butanal S (%)	Butanol S (%)	Crotyl Alcohol S (%)	Acetal S (%)
Cu_100_	98.1 ± 4.9	93.1 ± 4.7	3.9 ± 0.2	1.5 ± 0.1	1.6 ± 0.1
Pd_1_Cu_234_	99.3 ± 5.0	84.3 ± 4.4	13.3 ± 0.5	1.5 ± 0.1	1.0 ± 0.1
Pd_1_Cu_216_	99.7 ± 5.0	75.1 ± 3.8	22.0 ± 1.1	1.6 ± 0.1	1.3 ± 0.1
Pd_1_Cu_53_	99.0 ± 5.0	77.4 ± 4.1	20.8 ± 0.8	1.2 ± 0.1	0.6 ± 0.1
Pd_100_	99.2 ± 5.0	62.8 ± 3.1	37.2 ± 1.9	0.0	0.0
Blank	4.7 ± 0.2	0.0	0.0	0.0	100 ± 5.0
Al_2_O_3_	1.3 ± 0.1	0.0	0.0	0.0	100 ± 5.0

**Table 5:** Crotonaldehyde hydrogenation over the recycled catalysts. Reaction conditions: 7 h, 50 °C, 1.5 bar H_2_, 600 RPM, and 10 mg of catalyst.

Catalyst	Conversion (%)	Butanal S (%)	Butanol S (%)	Crotyl Alcohol S (%)	Acetal S (%)
Cu_100_^1^	98.1 ± 4.9	93.1 ± 4.7	3.9 ± 0.2	1.5 ± 0.1	1.6 ± 0.1
Cu_100_^2^	98.1 ± 4.9	94.4 ± 4.7	1.9 ± 0.1	1.6 ± 0.1	2.1 ± 0.1
Pd_1_Cu_216_^1^	99.7 ± 5.0	75.1 ± 3.8	22.0 ± 1.1	1.6 ± 0.1	1.3 ± 0.1
Pd_1_Cu_216_^2^	99.2 ± 5.0	73.7 ± 3.7	23.7 ± 1.2	1.5 ± 0.1	1.2 ± 0.1
Pd_1_Cu_53_^1^	99.0 ± 5.0	77.4 ± 4.1	20.8 ± 0.8	1.2 ± 0.1	0.6 ± 0.1
Pd_1_Cu_53_^2^	99.0 ± 5.0	77.8 ± 4.2	18.2 ± 0.6	1.6 ± 0.1	2.3 ± 0.1
Pd_100_^1^	99.7 ± 5.0	62.8 ± 3.1	37.2 ± 1.9	0.0	0.0
Pd_100_^2^	100.0 ± 5.0	65.3 ± 3.3	34.7 ± 1.7	0.0	0.0

Superscripts 1 and 2 indicate the catalyst cycle of testing, S—Selectivity (%).

## Data Availability

The data presented in this study are available on request from the corresponding authors.
